# Efficient *few-shot* machine learning for classification of EBSD patterns

**DOI:** 10.1038/s41598-021-87557-5

**Published:** 2021-04-14

**Authors:** Kevin Kaufmann, Hobson Lane, Xiao Liu, Kenneth S. Vecchio

**Affiliations:** 1 Department of NanoEngineering, UC San Diego, La Jolla, CA 92093 USA; 2 Tangible AI LLC, San Diego, CA 92037 USA; 3 Department of Healthcare Research and Policy, UC San Diego-Extension, San Diego, CA 92037 USA; 4 Materials Science and Engineering Program, UC San Diego, La Jolla, CA 92093 USA

**Keywords:** Characterization and analytical techniques, Microscopy

## Abstract

Deep learning is quickly becoming a standard approach to solving a range of materials science objectives, particularly in the field of computer vision. However, labeled datasets large enough to train neural networks from scratch can be challenging to collect. One approach to accelerating the training of deep learning models such as convolutional neural networks is the transfer of weights from models trained on unrelated image classification problems, commonly referred to as transfer learning. The powerful feature extractors learned previously can potentially be fine-tuned for a new classification problem without hindering performance. Transfer learning can also improve the results of training a model using a small amount of data, known as *few-shot* learning. Herein, we test the effectiveness of a *few-shot* transfer learning approach for the classification of electron backscatter diffraction (EBSD) pattern images to six space groups within the $\left( {4/m \overline {3} 2/m} \right)$ point group. Training history and performance metrics are compared with a model of the same architecture trained from scratch. In an effort to make this approach more explainable, visualization of filters, activation maps, and Shapley values are utilized to provide insight into the model’s operations. The applicability to real-world phase identification and differentiation is demonstrated using dual phase materials that are challenging to analyze with traditional methods.

## Introduction

Data science-based methods to materials development and analysis have gained great popularity in recent years^1–13^. Deep learning algorithms are of significant interest owing to their excellent performance without significant feature engineering, and the ubiquity of these methods will likely continue owing to the outperformance of systems directly designed by humans. While often difficult to assess how and why these ‘black box algorithms’ are capable of performing these tasks, these methods can provide significant value or spark new insights^14,15^. Application of these tools to image-based tasks in materials science has proved to be useful for classification^16–19^, segmentation^20–22^, and other objectives^23–25^. While deep learning provides significant opportunities for the advancement of materials science, robust application of these tools often requires much larger datasets than are typically available within the materials science community. Utilizing the knowledge deep neural networks have learned from other domains offers an opportunity to develop models in these domains, where data is sparse and further collection and labeling could be slow or tedious^26–28^.

Convolutional neural networks (CNNs) are a class of deep learning models that have proven effective for analyzing image data^29^. Before a CNN can be applied to a given task, it must learn to assign importance (learnable weights and biases) to various aspects of the image that maximize the network’s differentiation capabilities. Two general strategies exist for training convolutional neural networks: (1) the weights can be randomly initialized, or (2) the weights can be transferred from a model pre-trained on a separate but related task, often in a nearby domain with significantly more data, and then refined for the current objective. The first approach, commonly referred to as "training from scratch", requires a large dataset to avoid overfitting and perform robustly on new, real-world examples. The second approach, referred to as "transfer learning", can significantly reduce the number of training examples required, accelerate the training process, and retain or exceed the performance garnered by training from scratch^27,30–32^. The transfer learning method is motivated by the human ability to intelligently apply previously learned knowledge to solve new problems faster or with better solutions^33^. Despite the potential, knowledge transfer from a given source domain is not guaranteed to improve performance in the target domain and can in fact hinder performance^26,32^. Furthermore, one of the requirements to use this approach is that the images in the new domain must conform to the processed shape and structure determined at the outset of the previous training. For models pre-trained on ImageNet^34^, a library of over one million images labeled with one thousand classes, the expected input is usually 299 × 299 pixels with 3-channels (one each for RGB). When dealing with one-channel grayscale images, such as those typically collected from electron diffraction studies, the decision to use transfer learning necessitates the stacking of a single image into a pseudo-color image^31^.

The number of labeled images that can reasonably be collected must also be considered for the appropriate training and application of a CNN. Computer vision research has recently been motivated by children’s ability to learn novel visual concepts almost effortlessly after accumulating sufficient past knowledge^35^. In deep learning and computer vision, learning visual models of object categories has notoriously required tens of thousands of training examples^36^; however, recent research has demonstrated that it is possible to classify images accurately using relatively few labeled examples with the appropriate combination of pretraining of the CNN layers on unrelated image classification training sets^37,38^, adversarial or unsupervised learning^39,40^, network pruning^41^, and micro architecture tuning^42^. Once several categories have been learned the hard way, learning new categories should become more efficient. The increased efficiency allows for a lesser number of images to be used in training, referred to as a “*few shots*”.

Electron backscatter diffraction (EBSD) patterns (EBSPs) are an excellent case study for the use of *few-shot* transfer learning toward accelerating analysis of electron diffraction data. The scanning electron microscope (SEM)-based method involves the capture of 2D diffraction patterns produced from an incident electron beam scattering, diffracting, and escaping from a well-polished ‘bulk’ sample^43^. The collected diffraction patterns contain significant structural and chemical information and are similar to those collected in other techniques such as convergent beam electron diffraction (CBED)^44,45^. Despite the vast amount of information in the patterns, conventional EBSD has primarily focused on determining the three-dimensional orientation of individual grains in crystalline materials^43,46–48^. Furthermore, the commercial technique typically relies on Hough-based indexing with a look-up table of interplanar angles constructed from the set of selected reflectors for phases specified by the user^49^. This generally allows for phase differentiation of sufficiently distinct crystal structures^50–52^, but the process remains susceptible to structural misclassification^53–55^. Improvements to phase differentiation have been proposed and developed including dictionary indexing^56–59^, spherical indexing^60–62^, and more recently machine learning^63^. While each offers significant advantages over the Hough-based method, these tools continue to require assumptions about the number of phases and/or their structure. For example, the dictionary-based approach requires simulation of a “master” pattern for each potential phase and every experimental pattern is matched against a dictionary of simulated patterns for all potential phases^56^. The highest similarity match is selected as the most likely phase and orientation. Another available solution to phase differentiation is combining an electron backscattered pattern (EBSP) with information from energy dispersive X-ray spectroscopy (EDS) or wavelength dispersive X-ray spectroscopy (WDS)^64–66^. While these have been adopted commercially, the singular EBSP is still analyzed with the Hough-based method and an expert user is required to evaluate the plausibility of returned matches. Applications using hand-drawn lines overlaid on individual Kikuchi diffraction patterns have been developed for determining the Bravais lattice or point group; however, they remain limited by at least one of the following: analysis time per pattern, the need for an expert crystallographer, or necessitating multiples of the same diffraction pattern with different SEM settings^67^. Clearly, there remains a need for a rapid EBSP classification tool capable of functioning with one EBSD pattern while remaining suitable for even the most novice user.

Recently, the EBSD community has begun to explore the use of convolutional neural networks as a foundation for addressing modern EBSD challenges^16,68–70^. Despite the marked advances these works have made, the requirements for simulating^71^ or collecting experimental diffraction patterns from a significant number of materials and crystallographic orientations remains a limiting factor. In this work, we test the validity of *few-shot* transfer learning, starting from ImageNet weights, applied to classify EBSPs to one of six space groups. Herein, space group refers to the symmetry group of a configuration in three-dimensional space. We compare the time to converge, the individual kernels (weights of the neurons), activation maps, and the performance of models trained from scratch or by transfer learning. Though there has been considerable progress on interpretability of machine learning systems, mostly in the field of eXplainable AI (XAI)^72^, fully understanding the internal mechanisms of deep neural networks is still an area of active research^15,73^. Visualization of the most similar weights each model independently learned, their respective activation maps, and Shapley values can increase understanding of how the artificial intelligence models accomplishes its task. Building these XAI foundations can increase trust in the model’s later predictions and help identify when the prediction is incorrect. In addition to evaluating each model’s performance on holdout data, each model is also tested with 6900 EBSPs collected from a Ni_90_Al_10_ sample outside the training, testing, and validation data, and a space group map is generated from the individual classifications.

## Results and discussion

### Training metrics

**Figure 1:**
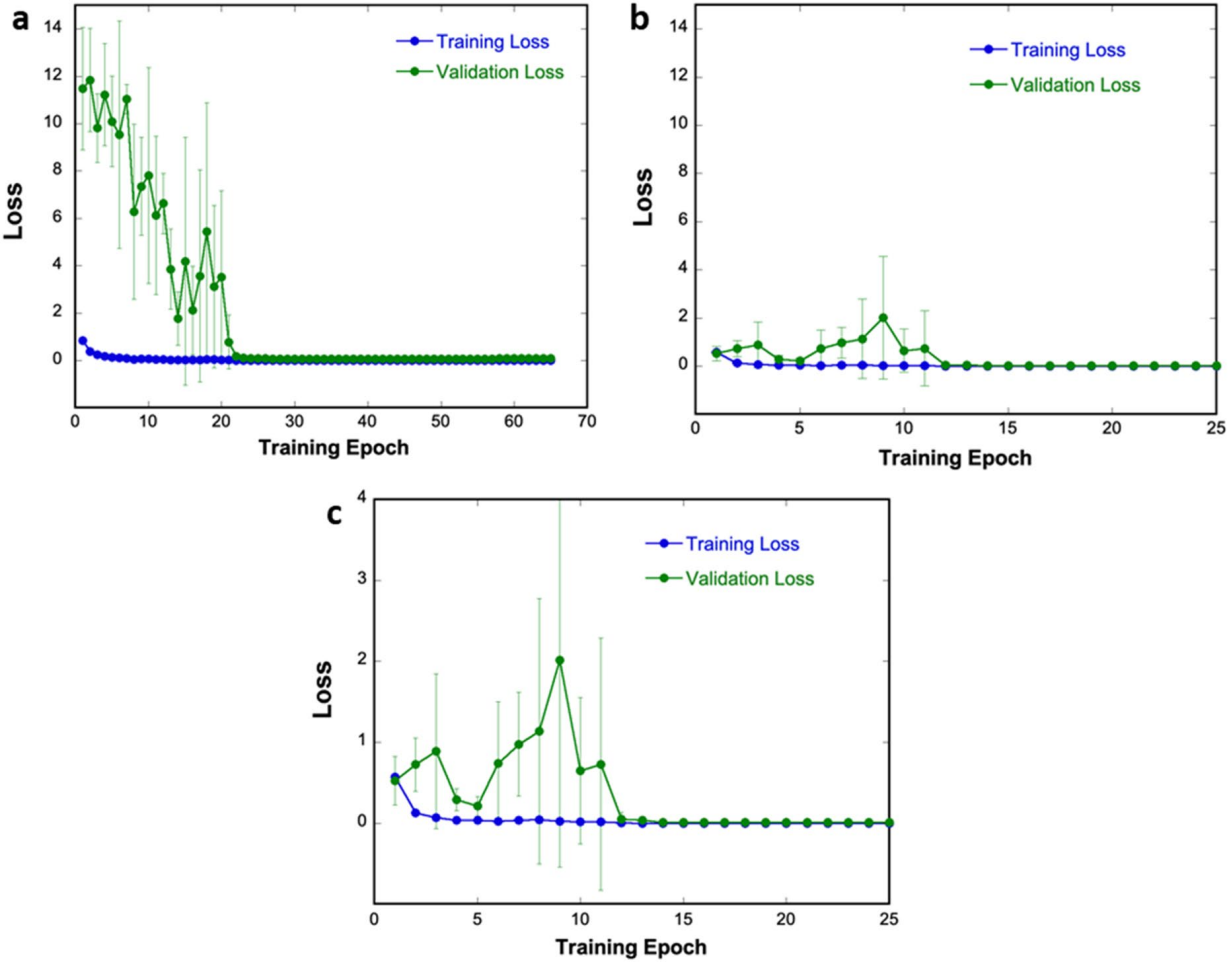
Training and validation history statistics for the two models. (**a**) The model trained from scratch using grayscale images. (**b**) The model trained starting from ImageNet weights using 3-channel stacked images. Data is plotted on the same y-axis as in (**b**). (**c**) The data in (**b**) for the transfer learning model plotted on a different scale. Error bars are one standard deviation from five trials per approach.

Each model is first trained until the validation loss converged using a small number of the available diffraction patterns from each material in the six available space groups. The training and validation loss were recorded at the end of each pass through the training set (i.e. an epoch) to monitor the model’s performance as the weights are updated. The validation loss is also monitored to determine when training should cease to prevent model underfitting or overfitting owing to a fixed number of training epochs. The loss function (categorical cross-entropy) is plotted in Supplementary Fig. S1 for reference. The goal of the neural network is to minimize the loss function by maximizing its prediction of the correct class. Figure 1a shows statistics for the training and validation loss for the model trained from scratch in grayscale. While the average training loss is low (0.84 ± 0.04) by the end of the first epoch, the average validation loss is observed to be 11.5 ± 2.5. In comparison, by initializing the model with weights learned from ImageNet, the average training and validation loss are both observed to be small (0.56 ± 0.02 and 0.52 ± 0.3, respectively) from the start (Fig. 1b). Figure 1c shows a magnified view of Fig. 1b to show the detailed training history. The improved starting loss, presumably owing to the strong filters learned in pre-training, also aids in rapid model convergence. Including the 15 epochs used to establish convergence, the model trained from scratch required 50 ± 12 epochs, while the transfer learning model required only 26 ± 3 epochs; this represents a twofold reduction in the average number of passes through the training set. Since each epoch with 2400 diffraction patterns requires two minutes on this hardware, a time savings of nearly an hour per training event is gained. With the amount of training data available to the CNN expected to grow, and therefore the time per epoch expected to increase, the time savings will become more pronounced.

### Performance on holdout data

The time saving advantages garnered by fine-tuning the weights of an existing model are only valuable if the model performs similarly well or exceeds the model trained from scratch. Table 1 shows the class-weighted *Precision* and *Recall* for the best performing model from each approach, for which further analysis into the internal operations will be studied. See Supplementary Tables S1 and S2 for a breakdown of *Precision* and *Recall* by space group for the transfer learned and trained from scratch models, respectively. Both metrics are improved for the model using transfer learning even though the training, validation, and test sets were held constant. This implies that the feature extractors learned from ImageNet are not only relevant to this new domain, but also at least as valuable as those learned from scratch. This likely results from the more general nature of the feature extractors necessary for optimal performance on ImageNet^74^. It is possible that increasing the size of the dataset used to train the model from scratch could increase its performance to near that of the *few-shot* transfer learning model; however, the cost to training time would be notable. For this study, it was also important to keep the dataset and its partitions fixed for more deterministic comparisons.

**Table 1:** Classification metrics.

	Precision	Recall
Trained from scratch	0.93	0.92
Transfer learning	0.97	0.96

The class-weighted average *Precision* and *Recall* on the test data for each model. Both metrics are improved using the transfer learning approach to training the model. The same test data was used to benchmark each model.

### Visualization

**Figure 2:**
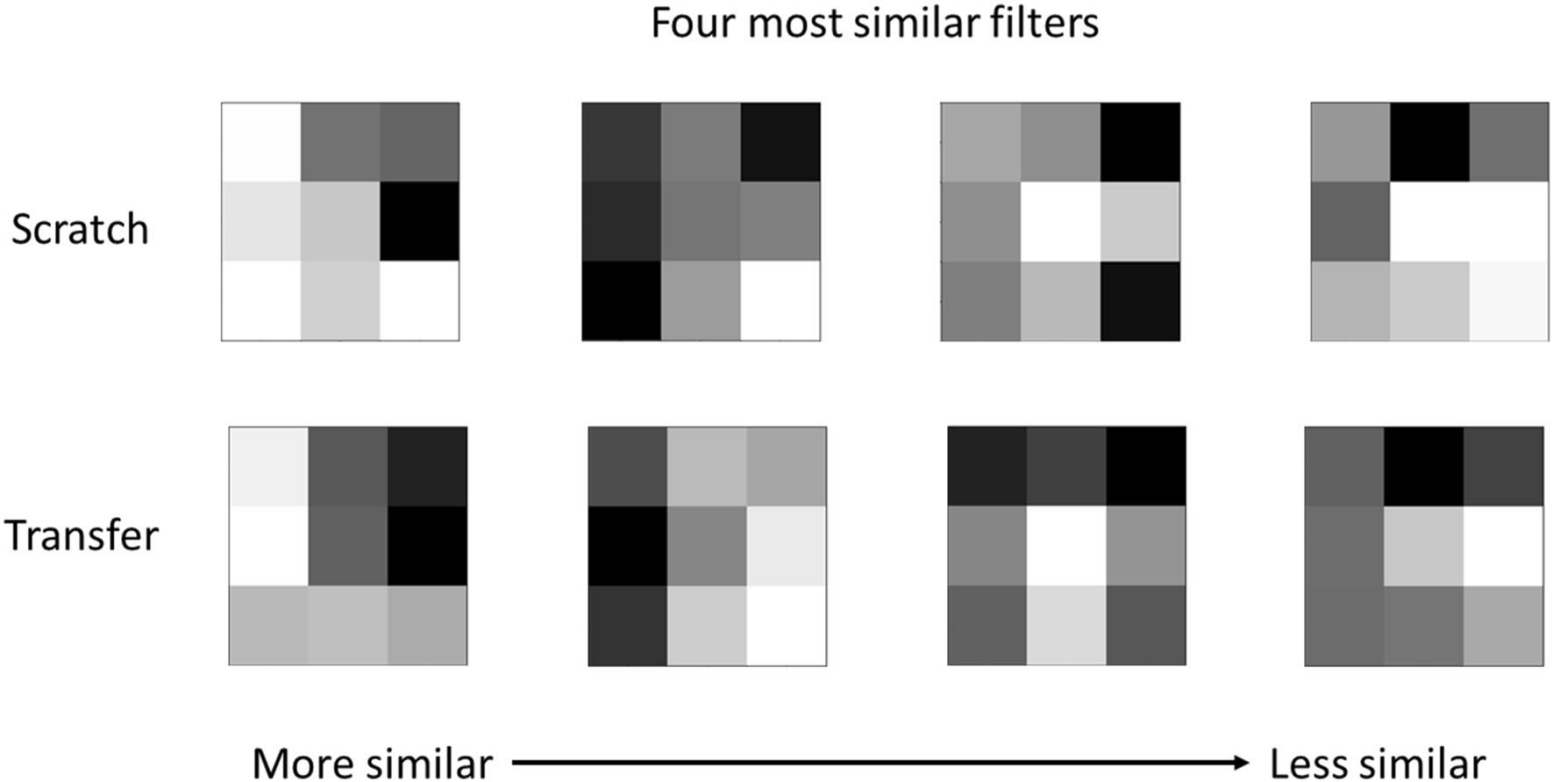
Kernel pairs with lowest Euclidean distance. Kernel pairs are grouped by column between the two models. The leftmost pair has the lowest Euclidean distance, with the kernels becoming less similar moving to the right.

Since deep neural networks are used in this study, meaningful information about the internal mathematical operations performed necessitates studying several aspects of the model’s inner workings. The first study involves visualization of the filters (or kernels) and corresponding feature maps. Filters and feature maps from the earliest layers in the model are most useful since deeper layers become more abstract. To identify corresponding filters in the two models, the Euclidean distance between all possible pairs of filters in a selected layer from the grayscale and pseudo RGB models were calculated and the similarity was ranked. The four most similar kernels between the two models in the first convolution layer are shown in Fig. 2. The pairs are grouped in columns between the model trained from scratch and using transfer learning. The independent co-learning of these filters suggest they are very valuable for feature tuning and selection on EBSPs. At this low-level in the model, the filters will predominantly be designed to identify edges at various orientations. The next layer will likely combine those edges into corners and small points, and the subsequent layers will figure out larger and larger shapes/features, such as the number of and relative angles between Kikuchi lines. Two of these four filters are recognizable as classical edge detection operators. The second filter has converged close to the x-direction Sobel edge detection operator^75^ and the third filter resembles the Gabor filters with theta ${\uptheta } = 135^\circ$. Visualization of the feature maps resulting from these four filters can provide insight into their function.

**Figure 3:**
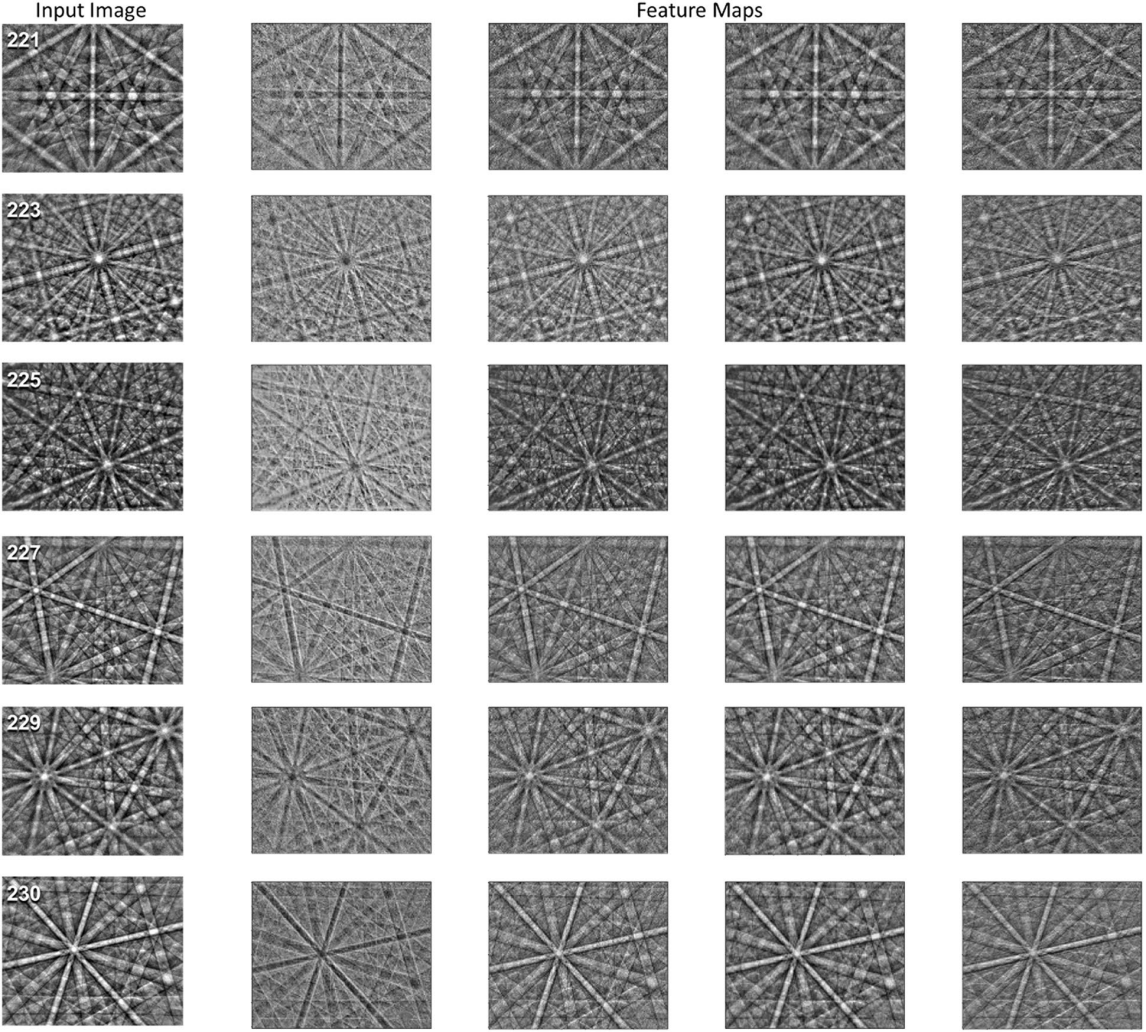
Selected feature maps from the first convolution layer. Feature maps extracted from the grayscale model corresponding to the filters shown previously in Fig. 2. One input image per space group is shown. From top to bottom, the six space groups are $Pm\overline{3}m$, $Pm\overline{3}n$,$Fm\overline{3}m$,$Fd\overline{3}m$,$Im\overline{3}m$, and $Ia\overline{3}d$.

Figure 3 shows the result of each filter from Fig. 2 being individually convolved over an input image from the six space groups studied. All feature maps shown are from the grayscale model. The first filter, and therefore the most similar filter between the two models, is primarily observed to perform an inversion on the input image. As a result, the band edges in each image are activated (white regions) and become more distinct. It is reasonable to speculate that deeper parts of the network extract the angle and relative locations of these intersections, a function that was also suggested in Ding et al.’s recent work^70^. The fourth filter is observed to have normalized the contrast of the input image, perhaps to reduce the effects of atomic scattering factors (Z-contrast) observed in prior work^68^. Further analysis of the class-specific feature importance can further improve understanding of the neural network’s methodology.

### Feature importance

**Figure 4:**
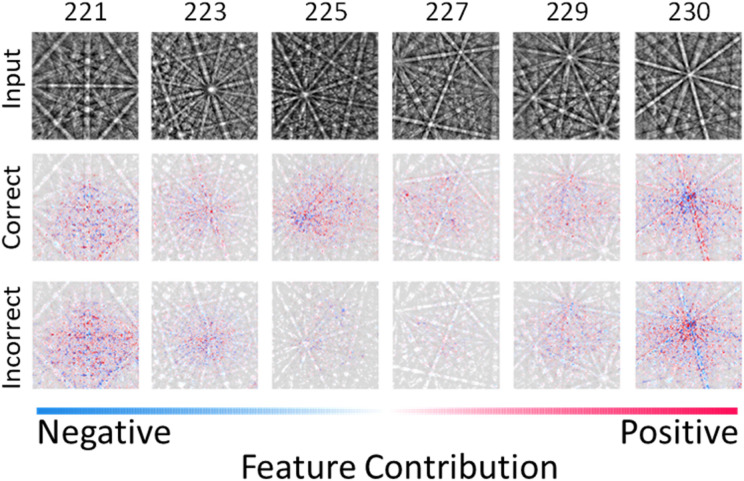
Visual explanation of feature contributions. Shapley values are computed for each input image to gauge the importance of features in the EBSPs. The first row is the raw input image. The second row corresponds to the Shapley values for the correct prediction. Row three corresponds to the first incorrect classification as ranked by softmax probability. From left to right, the six space groups are $Pm\overline{3}m$ $Pm\overline{3}n$,$Fm\overline{3}m$,$Fd\overline{3}m$,$Im\overline{3}m$, and $Ia\overline{3}d$.

Measurement and visualization of feature importance is another method to increase understanding of the deep neural network. This type of analysis is performed to help determine whether one should trust a prediction and why. There are a number of techniques available for CNNs including Gradient-weighted Class Activation Mapping (Grad-CAM)^76^, activation maximization^77^, LIME^78^, and Shapley values^79^. Several of those listed have been effectively demonstrated in other works involving EBSD patterns^16,17,68,70^; however, this is the first to use Shapley values. In game theory, Shapley values are a solution to fairly distributing the gains and costs of several actors working in coalition. By definition, each Shapley value is the average expected marginal contribution of one actor after all possible combinations have been considered. In this case, the actors are the features in the images. Essentially, the Shapley value is the average expected marginal contribution of one actor after all possible combinations have been considered. While not perfect, this has proven a fair approach to allocating value in a variety of fields^80–82^. The results of this analysis, further described in the Methods section, are shown in Fig. 4. Refer to Supplementary Fig. S2 for a demonstration of SHAP analysis on handwritten numbers.

The previously unseen input images are shown in the top row, and as semi-transparent grayscale backings behind each of the explanations. After random selection of the input image, it was verified that the model correctly identified the space group (thus pseudo-random selection). The middle row in Fig. 4 corresponds to the explanations for the correct prediction, while the bottom row displays the explanations for the next most likely (i.e. incorrect) space group. All 6 Shapley explanations ordered by most to least likely space group for each input are displayed in Supplementary Fig. S3. The positive (red) and negative (blue) contributions to each prediction are primarily at diffraction maxima (e.g. zone axes), band intersections, and outlining band edges. This lends credence that the model is indeed utilizing information grounded in the physics of EBSD. The clustering of Shapley values near zone axes is further reaffirming given the abundance of information and their role in classical diffraction pattern indexing^83^.

### Performance in practice

**Figure 5:**
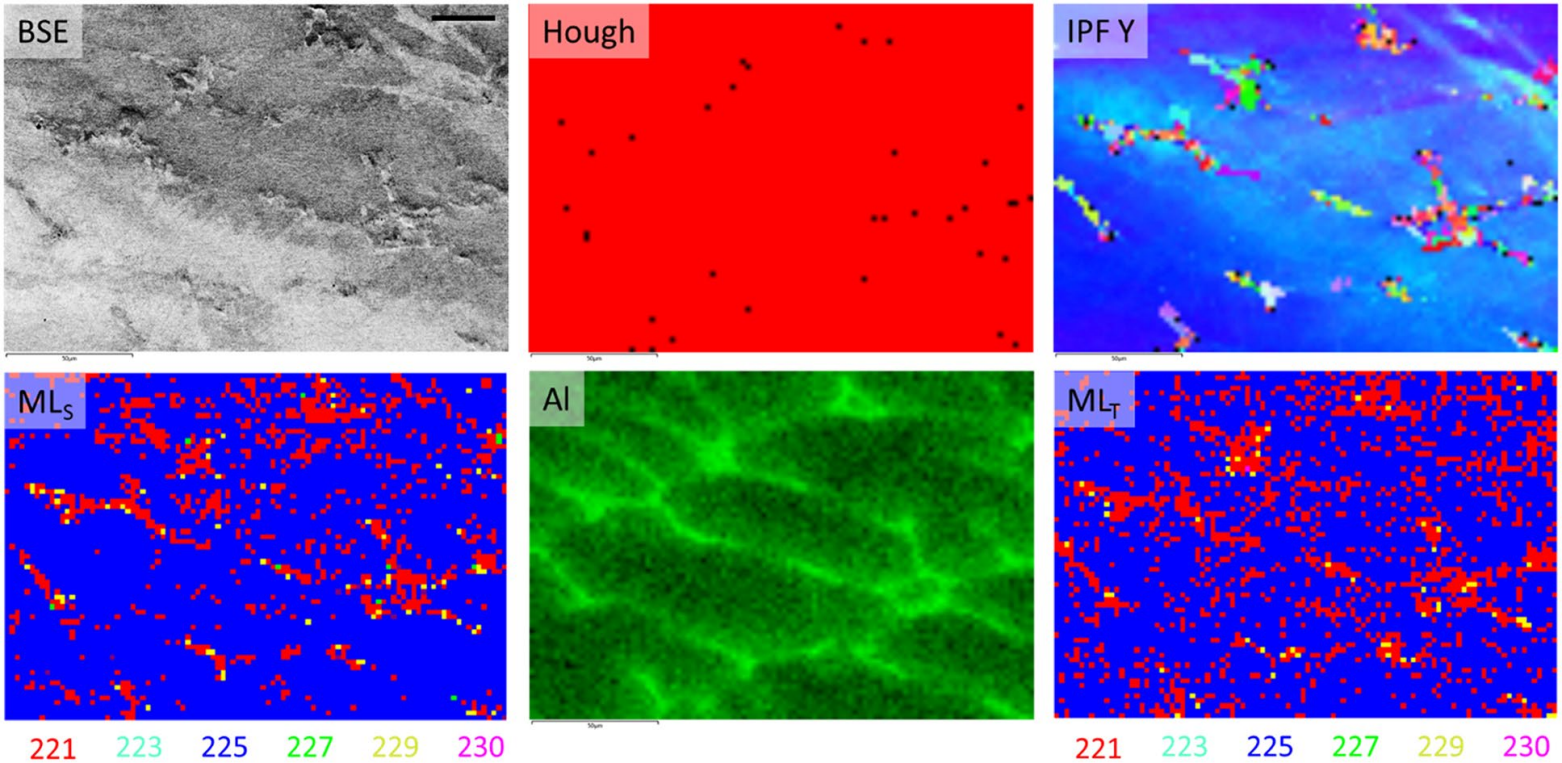
Phase mapping a dual-phase sample. Top left shows a backscattered electron image of the area to be mapped. The Ni_3_Al precipitates appear raised in the Ni-rich matrix. Top middle shows the Hough transform-based phase map. Red pixels are identified as Ni or Ni_3_Al with equal certainty, while black pixels were not solved. Top right is the inverse pole figure map in the Y-direction. Bottom left is the phase map produced using the predictions from the grayscale CNN. Bottom middle shows the aluminum EDS map. Bottom right is the phase map produced using the predictions from the *few-shot* transfer learning approach. There are 6900 total EBSPs (pixels). Scale bar = 25 µm.

It is also of importance to demonstrate and compare the efficacy of both models in a real-world context, not only the patterns set aside for testing. In this case, the EBSD mapping of a dual phase sample serves to demonstrate that both model training strategies are capable in situations where the diffraction patterns are not collected from an ideal, single-phase material. Figure 5 top-left shows a backscattered electron image of a Ni_90_Al_10_ (wt%) sample containing a Ni-rich matrix (space group 225) along with Ni_3_Al precipitates (space group 221) appearing raised from the surface. It is of importance to note that the model has not yet encountered a solid-solution phase such as the Ni with Al matrix present in this sample. The Al content in the Ni matrix was determined to be 19.7% ± 0.53% (at%). The Al chemistry of the Ni_3_Al precipitates was determined to be 25.7% ± 0.74% (at%). While partially selected for this reason, the material was primarily chosen since space groups 221 and 225 have two of the lowest F1-scores in each model and can readily be produced in this singular sample. Furthermore, it was important that the phases could be differentiated visually and easily to the reader, such as with EDS maps. While this means the phases could potentially be differentiated if the EBSD operator assigned reference chemistries to the phases in advance of collecting the EBSPs, the purpose of this demonstration is really to compare the transfer learning and trained from scratch models’ abilities to identify the space group without further information. For the most complete phase identification in EBSD (i.e. lattice parameters), multiple analysis methods (e.g. XRD and EDS) may need to be employed.

The Hough-based phase map in Fig. 5 is a representative image of the expected results from commercial systems. The phase map consists of entirely one phase (shown in red) and Oxford Aztec software is observed to predict Ni and Ni_3_Al with an equal number of bands and mean angular deviation (MAD), effectively a combined measure of certainty, for each diffraction pattern. It is the order of phase selection by the user in Oxford Aztec software that ultimately determines whether Ni or Ni_3_Al is selected in this case. Example diffraction patterns from each phase are shown in Supplementary Fig. S4. Black pixels remain unindexed by the Hough method, typically a result of poor diffraction pattern quality. The inverse pole figure (IPF) in the Y-direction is provided along with the EDS map for aluminum to elucidate where the Ni_3_Al is expected to be found within the Ni matrix.

The last two images in Fig. 5 show the phase maps produced by the scratch (ML_S_) and *few-shot* transfer learned (ML_T_) models. Over a statistical number of diffraction patterns, the two models are expected to produce similar answers with some variance, although the test set would suggest that the transfer learning model will generally outperform. Comparing the results with the Al EDS map and IPF Y, both models perform well at identifying the space group of each EBSP with a low false positive rate for the four space groups known not to be present. There are almost certainly some errors with regard to the EBSPs classified as space group 221 ($Pm\overline{3}m$) or 255 ($Fm\overline{3}m$), the *Precision* and *Recall* of each model alert the user of this in advance; however, the results over these 6900 EBSPs demonstrate the improvement over the Hough-based approach and, more importantly for this study, the robustness of a *few-shot* transfer learning approach.

The number of EBSPs each model classifies to the available space groups is tallied in Table 2. A total of 6900 diffraction patterns were individually identified by each neural network without any other information provided. The transfer learning model is observed to have a reduced misclassification rate to the space groups 227 ($Fd\overline{3}m$), 229 ($Im\overline{3}m$), and 230 ($Ia\overline{3}d$) in this phase map. The largest difference between the two models is for the diffraction patterns classified to space group 221; the class with the lowest *Precision* for each of the two models. The total difference of 517 diffraction patterns only equates to 7% of the total diffraction patterns; well within reason and the expected margin of error between the two models, particularly between these two space groups for the current models. Of those 517, only 480 of these predictions differ between space groups 221 and 225, the other 37 differences are due to false positives (i.e. 227, 229, and 230) in the model trained from scratch. The phase fraction of Ni_3_Al likely lies somewhere between what is predicted by these two models. The results of this comparison also suggests that there is future opportunity to construct an approach leveraging Bayesian deep learning or an ensemble of (i.e. at least two) individually trained models making individual classifications combined with model averaging (e.g. voting) to reduce variance, provide insight into overall uncertainty, and identify when “no solution” is an appropriate answer^84–86^.

**Table 2:** Tabulated predictions for the dual-phase sample.

	221	223	225	227	229	230
Scratch	984	0	5822	10	72	12
Transfer	1501	0	5342	0	56	1
Difference	517	0	(480)	(10)	(16)	(11)

The number of patterns classified to each space group by the respective model. The difference is calculated by subtracting the number predicted by the model trained from scratch from the number predicted by the *few-shot* transfer learning model. Parentheses denotes the transfer learning model predicted fewer EBSPs belonging to the respective class. From left to right, the six space groups are $Pm\overline{3}m$, $Pm\overline{3}n$,$Fm\overline{3}m$,$Fd\overline{3}m$,$Im\overline{3}m$, and $Ia\overline{3}d$.

Thus, a *few-shot* transfer learning approach to classifying electron backscatter diffraction patterns is an attractive method for leveraging the knowledge a deep neural network has attained in a previous context. The convolutional neural network-based approach to diffraction pattern classification is advantageous in that it requires little or no a-priori knowledge of the phases in a new sample and can readily be improved or expanded to new classes with the inclusion of new data. The similarity of EBSD patterns to those from techniques such as CBED suggests the *few-shot* transfer learning approach could also apply and potentially be more beneficial given the slower rate of data collection with other electron diffraction methods. Limitations of the current models exist in the number of space groups currently differentiable and the “black box” nature of neural networks. The number of space groups the model can learn to differentiate can be continuously expanded as more data becomes available for training. One of the goals of this work is to discern whether the *few-shot* transfer learning approach can be used to reduce the amount of data necessary for robust expansion to all 230 space groups or other diffraction pattern classification tasks. Indeed, we find that this approach does not hinder the model’s performance on holdout or entirely new data and offers accelerated training time. While it can be difficult to precisely determine how the CNN performs this task, recent advances in eXplainable AI (e.g. SHAP) provides tools for developing insight and trust in the model’s predictions. The combination of ease of scaling, flexibility of the framework, and ability to assess aspects of a model’s decision process support the utilization of CNNs and *few-shot* transfer learning as another tool for phase differentiation and symmetry identification in electron diffraction.

## Materials and methods

### Materials

Eighteen different single-phase materials, comprising 6 of the 10 space groups within the $\left( {4/m\overline{ 3} 2/m} \right)$ point group, were selected for training the space group classification CNN. Suitable samples for the remaining 4 space groups could not be obtained. The six space groups are $Pm\overline{3}m$, $Pm\overline{3}n$,$Fm\overline{3}m$,$Fd\overline{3}m$,$Im\overline{3}m$, and $Ia\overline{3}d$. Numerically, these are space groups 221, 223, 225, 227, 229, and 230. Space groups 221 and 223 are primitive cubic, 225 and 227 are face centered cubic, and 229 and 230 are body centered cubic. Each of the six space groups share the threefold rotary inversion necessary for inclusion in the $\left( {4/m\overline{ 3} 2/m} \right)$ point group. Supplementary Table S3 details the similarities and differences between the symmetry operations of the six space groups. The materials were FeAl, NiAl, Ni_3_Al, Fe_3_Ni, Cr_3_Si, Mo_3_Si, Ni, Al, NbC, TaC, TiC, Si, Ge, W, Ta, Fe, Al_4_CoNi_2_, and Al_4_Ni_3_. These materials were of low texture, typically less than 2 times random in any direction. Refer to Kaufmann et al. for the distributions of orientation, band contrast, and mean angular deviation for these samples^69^.

A dual-phase material known to challenge Hough-based EBSD was fabricated to demonstrate and compare the capabilities of each CNN training approach. An additional constraint for the material selected was that the two space groups be identifiable within an EDS map, even though this meant an operator could force the Hough-based method to differentiate the phases by chemistry if they knew the phases in advance. An ingot of Ni_90_Al_10_ (wt%) was arc melted and processed via hot rolling at 600 °C to 45% reduction in thickness followed by aging at 600 °C for 4 h and air cooled. X-ray diffraction (XRD) using a Rigaku Miniflex X-ray Diffractometer with a 1D detector, a step size of 0.02°, 5° per minute scan rate, and Cu Kα radiation (wavelength λ = 1.54059 Å) was performed to confirm the existence of phases belonging to space groups 221 ($Pm\overline{3}m$) and 225 ($Fm\overline{3}m$) (Supplementary Fig. S5).

### Electron backscatter diffraction pattern collection

EBSD patterns (EBSPs) were collected as previously described in Kaufmann et al*.*^68^. Diffraction patterns were collected using a Thermo Scientific (formerly FEI) Apreo scanning electron microscope (SEM) equipped with an Oxford Symmetry EBSD detector utilized in high resolution (1244 × 1024) mode. The geometry of the setup was held constant as follows. The working distance was 18.1 mm ± 0.1 mm. Oxford Aztec software was used to set the detector insertion distance to 160.2 and the detector tilt to -3.1. The imaging parameters were 20 kV accelerating voltage, 51 nA beam current, 0.8 ms ± 0.1 ms dwell time, and 30-pattern averaging. The Hough indexing parameters were 12 Kikuchi bands, a Hough resolution of 250, and band center indexing.

After collecting high resolution EBSPs from each material, all patterns collected were exported as tiff images. The images were resized for the CNN using the resize function in scikit-image. All collected data for each material was individually assessed by the neural network, and the collection of images for each sample may contain partial or low-quality diffraction patterns, which could decrease the accuracy of their identification. The test data was not filtered to better assess the model as it would be applied in practice.

### Neural network architecture

The well-studied convolutional neural network architecture Xception^87^ was selected as the basis architecture for fitting a model that determines which space group a diffraction pattern originated from. The Xception architecture was used without modifications for training the model from scratch and the transfer learning process to facilitate comparison of the training metrics, performance, and internal workings. Selection of this network was partially based on Xception or derivatives of Xception being used previously in the EBSD community^16,68–70^. Xception is also a standard model with ImageNet weights readily available in deep learning APIs such as Keras^88^. A schematic of the convolutional neural network operating on an EBSP is provided in Fig. S6. Due to space constraints, only the resultant feature maps from selected convolutional layers are shown after image input and before the 2048-dimensional vector. For a complete description of the Xception architecture, please refer to Fig. 5 in Xception: Deep Learning with Depthwise Separable Convolutions^87^.

### Neural network training

For both the transfer learning and from scratch approaches, training was performed using 400 diffraction patterns per space group. The diffraction patterns supplied at training were evenly divided between the number of materials per space group that the model had access to during training. For example, if the model was given two materials of the same space group during training, 200 diffraction patterns per material were made available. The validation set contained 100 diffraction patterns per space group, equivalent to the standard 80:20 train/validation split. The validation set was only used to monitor the training progress and model convergence. The test set contains the rest of the patterns (a total of 145,453 images; refer to Supplementary Table S1 for class distribution) that were not used for training or validation. The images selected for training, testing, and validation were the exact same for transfer learning and for from scratch learning.

Model hyperparameters were selected or tuned as follows. Adam (adaptive moment estimation) optimization with a learning rate of 0.001^89^, and a minimum delta of 0.001 as the validation loss were employed for convergence criteria. Adam is chosen for its ability to work well with little hyperparameter tuning, relatively low memory requirements, and its ability to smooth the steps of gradient descent using momentum. Monitoring of validation loss, i.e. early stopping criteria, was employed instead of a fixed number of epochs to allow both models the necessary epochs to converge while keeping the risk of overfitting to the training data low. The patience criteria for validation loss convergence was set to 15 epochs to allow for sufficient certainty that the model had converged and was unlikely to meaningfully improve. The weight decay was set to 1e^−5^ following previous optimization work^87^. The CNNs were implemented with TensorFlow^90^ and the Keras API^88^ and model training was performed using an NVIDIA Titan V.

### Diffraction pattern classification

Each diffraction pattern collected, but not used in training (> 140,000 images), was evaluated in a random order by the corresponding trained CNN model without further information. The output classification of each diffraction pattern was recorded, saved in a (.csv) file, and tabulated. *Precision* and *Recall* were calculated for each material and each space group using Scikit-learn^91^. *Precision* (Eq. 1) for each class (e.g. 230) is defined as the number of correctly predicted images out of all photos predicted to belong to that class (e.g. 230). *Recall* (Eq. 2) is the number of correctly predicted images for each class divided by the actual number of images for the class. F1-score is the weighted harmonic of the *Precision* and *Recall* and is particularly valuable in situations where the number of test images per class is variable. A high F1-score means the model has low false positives and low false negatives.

1 $$Precision = \frac{true \,positives}{{true \,positives + false \,positives}},$$

2 $$Recall = \frac{true \,positives}{{true \,positives + false\, negatives}}.$$

### Neural network insight

Comparisons between the resultant models from pseudo RGB transfer learning and training the model from scratch offer an opportunity to understand how the CNNs go about their given task. Visualization techniques are implemented using the keras-vis package v0.4.1^77^. Filters from the first layer of each model are extracted and plotted as 3 × 3 matrices with matplotlib^92^. The first layer was targeted since the earliest filters represent lower level features such as colors and edges. The Euclidean distance between the individual filter arrays in each model was computed using the NumPy linear algebra toolbox to compute L2 norms^93^ and the four most similar learned filters between the two models were identified. The outputs (a.k.a. feature maps) of the first layer corresponding to these four filters are also extracted to examine the activations of the two approaches. The feature maps from earlier convolution layers are more useful since deeper layers operate in feature space and are therefore more difficult to understand^29,70^. Lastly, Shapley values, deeply rooted in game theory^94,95^, are estimated using the DeepSHAP tools in SHAP^96^. SHAP uses a distribution of background samples, approximates the model with a linear function between each background data sample and the current input to be explained, and assumes the input features are independent to compute approximate SHAP values. The sum of the SHAP values equals the difference between the expected model output (averaged over the background dataset) and the current model output. One hundred images per space group were used as background samples in conjunction with one new input image per space group. The total number of diffraction patterns used as a background follows the SHAP software protocols. When plotted as an overlay, red pixels represent positive SHAP values that increase the probability of the class, while blue pixels represent negative SHAP values that reduce the probability of the class. An example of SHAP analysis on handwritten digits from the MNIST database^97^ is shown in Supplementary Fig. S2. For a given image, the presence and absence of features that positively correlate with a class are shown in red, while negative correlations are shown in blue. As an example, in the image of a ‘four’ the lack of a connection on top makes it a four instead of a nine. Combined, these insights into the operations of the neural network can further substantiate the validity of the transfer learning approach, increase trust by better understanding the model’s methods, and provide indications in cases where the model is incorrect about future predictions.

## Supplementary Information

Supplementary Information.

## Data Availability

The datasets generated during and/or analyzed during the current study are available from the corresponding author on reasonable request. The code will be available in the GitHub repository https://github.com/krkaufma/EBSD_transfer_learning and in the online Zenodo repository (https://doi.org/10.5281/zenodo.3564937)^98^.
