# Supplementary material for: Efficient few-shot machine learning for classification of EBSD patterns
Source: Sci Rep. 2021 Apr 14;11:8172. doi: 10.1038/s41598-021-87557-5 (PMC8046977; doi:10.1038/s41598-021-87557-5)
Supplement: Supplementary file 1 — Supplementary Information. [file 41598_2021_87557_MOESM1_ESM.pdf]

Supplementary Materials for

Efficient few-shot machine learning for classification of EBSD patterns

Kevin Kaufmann[1], Hobson Lane[2,3], Xiao Liu[4], and Kenneth S. Vecchio[1,4*]

Correspondence to: kvecchio@eng.ucsd.edu

This PDF file includes:

Tables S1 to S3

Figs. S1 to S6

Table S1. Classification metrics by space group for the transfer learning model. *Precision* is the number of patterns correctly identified to a class divided by the total number of patterns identified as that class. *Recall* is the percentage of patterns in a space group that were correctly identified. F1-score is the weighted harmonic mean of *Precision* and *Recall*. Test EBSPs is the total number of diffraction patterns in the test set for the given class.

**Space Group**	***Precision***	***Recall***	**F1-score**	**Test EBSPs**
**221**	0.89	0.98	0.93	25,955
**223**	1.00	1.00	1.00	23,248
**225**	0.99	0.94	0.96	62,277
**227**	0.95	1.00	0.97	10,978
**229**	0.98	0.96	0.97	19,374
**230**	0.87	0.99	0.93	3,621

Table S2. Classification metrics by space group for the model trained from scratch. Precision is the number of patterns correctly identified to a class divided by the total number of patterns identified as that class. Recall is the percentage of patterns in a space group that were correctly identified. F1-score is the weighted harmonic mean of *Precision* and *Recall*. Support is the total number of images in the test set.

**Space Group**	***Precision***	***Recall***	**F1-score**	**Support**
**221**	0.83	0.94	0.88	25,955
**223**	0.99	0.99	0.99	23,248
**225**	0.98	0.87	0.92	62,277
**227**	0.83	1.00	0.91	10,978
**229**	0.89	0.93	0.91	19,374
**230**	0.92	0.98	0.95	3,621

Table S3. Comparison of the symmetry elements that describe each space group. For each space group in this work, the Bravais lattice and primary, secondary, and tertiary symmetry operations are detailed. Within the $(4/m\,\overline{3}\,2/m)$ point group, the secondary symmetry element is always a 3-fold rotary inversion, while the primary and tertiary symmetry operations vary.

**Space Group Number**	**Space Group Name**	**Bravais Lattice**	**Symmetry and [Direction]**		
			Primary [100]/[010]/[001]	Secondary [111]	Tertiary [110]
221	$Pm\overline{3}m$	Primitive	Crystal is mapped onto itself by reflecting across a mirror plane perpendicular to [100]/[010]/[001]	Crystal is mapped back onto itself by a 3-fold rotary inversion in the [111]	Crystal is mapped onto itself by reflecting across a mirror plane perpendicular to [110]
223	$Pm\overline{3}n$	Primitive	Crystal is mapped onto itself by reflecting across a mirror plane perpendicular to [100]/[010]/[001]	Crystal is mapped back onto itself by a 3-fold rotary inversion in the [111]	Crystal is mapped back onto itself by a diagonal glide in the [110]
225	$Fm\overline{3}m$	Face Centered	Crystal is mapped onto itself by reflecting across a mirror plane perpendicular to [100]/[010]/[001]	Crystal is mapped back onto itself by a 3-fold rotary inversion in the [111]	Crystal is mapped onto itself by reflecting across a mirror plane perpendicular to [110]
227	$Fd\overline{3}m$	Face Centered	Crystal is mapped back onto itself by a diamond glide in the [100]/[010]/[001] direction	Crystal is mapped back onto itself by a 3-fold rotary inversion in the [111]	Crystal is mapped onto itself by reflecting across a mirror plane perpendicular to [110]
229	$Im\overline{3}m$	Body Centered	Crystal is mapped onto itself by reflecting across a mirror plane perpendicular to [100]/[010]/[001]	Crystal is mapped back onto itself by a 3-fold rotary inversion in the [111]	Crystal is mapped onto itself by reflecting across a mirror plane perpendicular to [110]

230	$Ia\bar{3}d$	Body Centered	Crystal is mapped onto itself by gliding half the lattice vector in the [100]/a direction	Crystal is mapped back onto itself by a 3-fold rotary inversion in the [111]	Crystal is mapped back onto itself by a diamond glide in the [110]

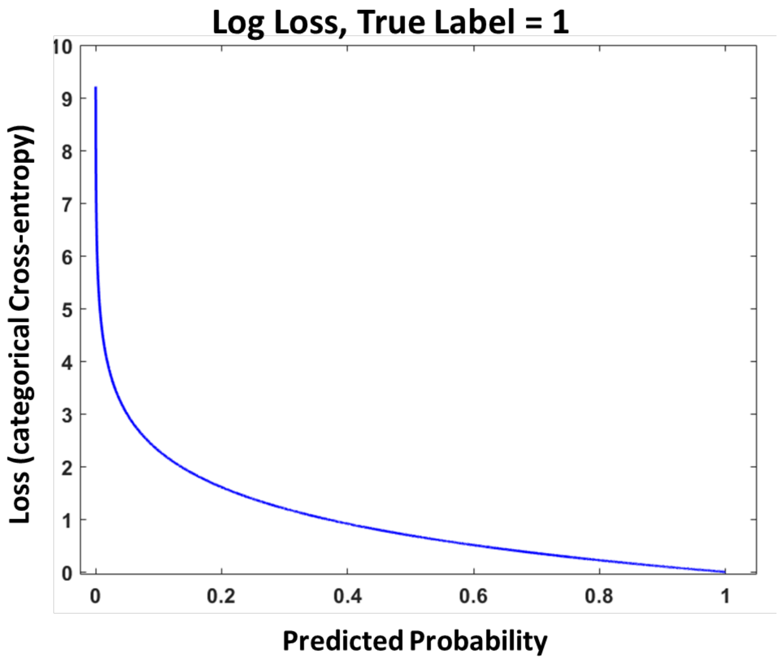

Figure S1. **The categorical cross-entropy loss function.** Categorical cross-entropy increases as the predicted probability diverges from the actual label.

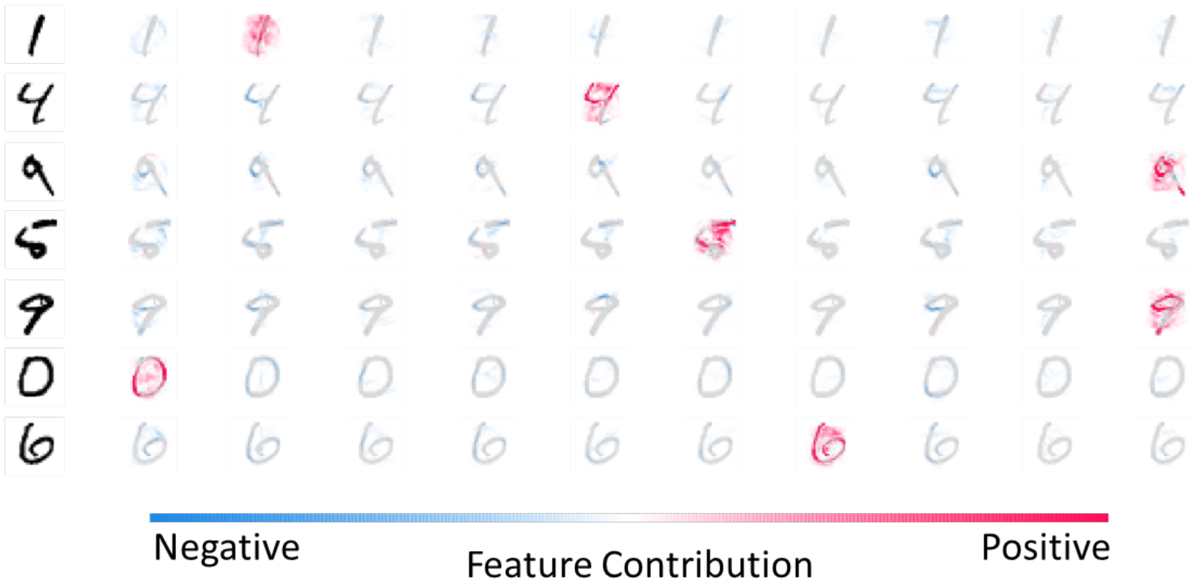

Figure S2. **Shapley value analysis for handwritten digits.** Shapley values are computed for several new images to gauge the importance of features (present or not) in predicting the handwritten number. The first column is the raw input image. The ensuing columns correspond to the Shapley values for each possible class in 0-9 order.

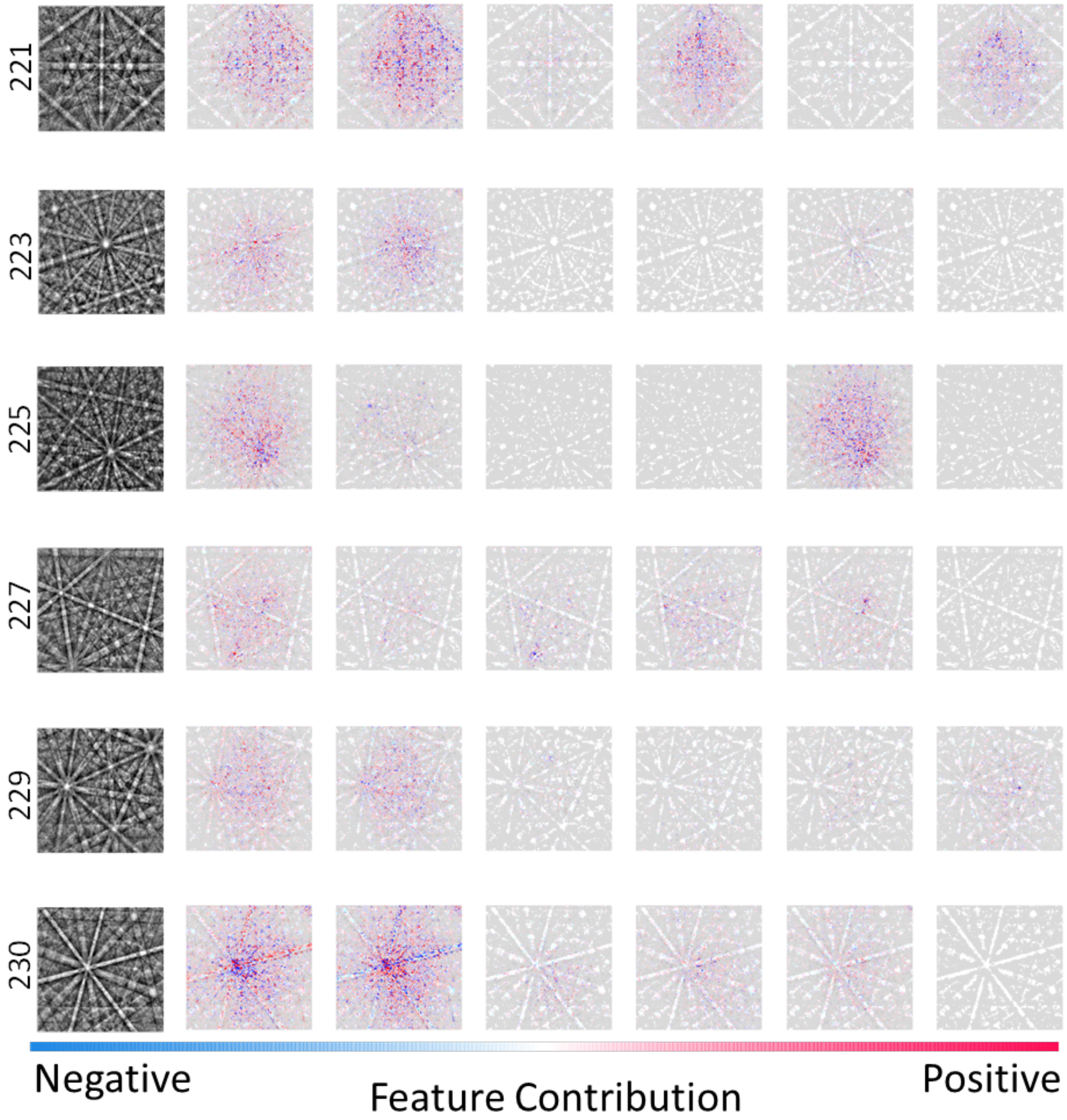

Figure S3. **Visual explanation of feature contributions.** Shapley values are computed for each input image to gauge the importance of features in the EBSPs. The first column is the raw input image. The second column corresponds to the Shapley values for the correct prediction. Columns three through seven correspond to incorrect classifications in softmax order.

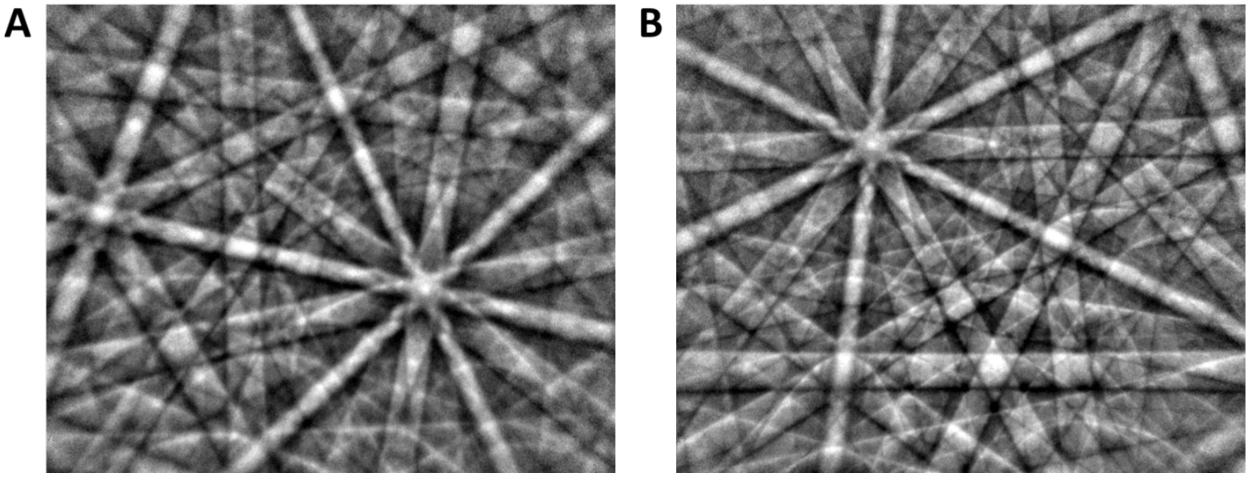

Figure S4. **Example EBSPs from the $Ni_{90}Al_{10}$ sample.** An EBSP from (A) the Ni-rich matrix and (B) the Ni_3Al precipitates are shown as examples.

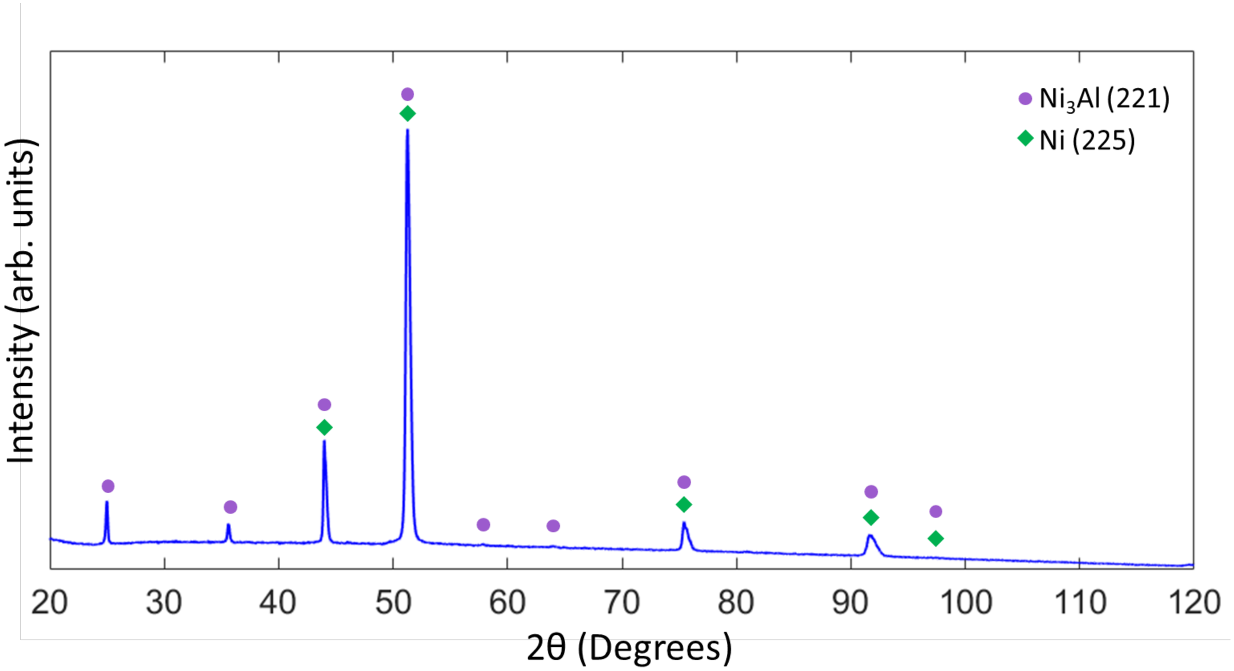

Figure S5. **XRD Pattern for the $Ni_{90}Al_{10}$ sample.** The peaks for Ni_3Al (space group 221) and Ni (space group 225) are labeled with purple circles and green diamonds, respectively.

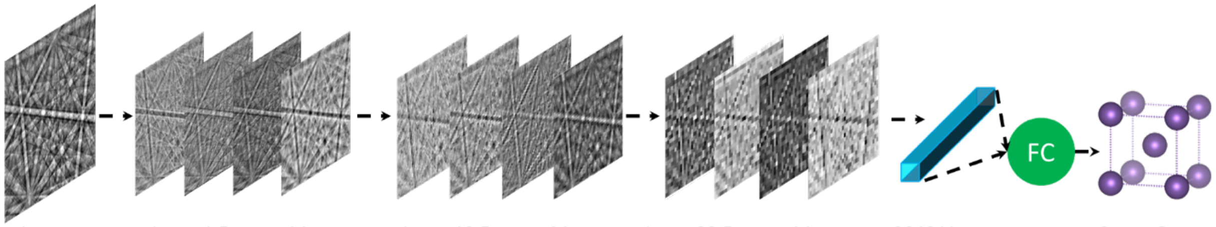

Figure S6. **Schematic of Neural Network Operating on an EBSP.** Individual EBSPs are input to the neural network wherein a series of mathematical operations extract features learned during the training or fine-tuning process. Selected feature maps obtained from several layers are shown. Eventually, the input image is reduced to a 2048-dimensional vector that is passed into a series of fully connected layers (FC) followed by multi-class logistic regression.
